# Design of a novel intelligent computing framework for predictive solutions of malaria propagation model

**DOI:** 10.1371/journal.pone.0298451

**Published:** 2024-04-18

**Authors:** Kottakkaran Sooppy Nisar, Muhammad Wajahat Anjum, Muhammad Asif Zahoor Raja, Muhammad Shoaib

**Affiliations:** 1 Department of Mathematics, College of Science and Humanities in Alkharj, Prince Sattam Bin Abdulaziz, University, Alkharj, Saudi Arabia; 2 Department of Mathematics, COMSATS University Islamabad, Attock Campus, Pakistan; 3 Future Technology Research Center, National Yunlin University of Science and Technology, Douliou, Yunlin, Taiwan, R.O.C; 4 Yuan Ze University, AI Center, Taoyuan, Taiwan; Newcastle University, UNITED KINGDOM

## Abstract

The paper presents an innovative computational framework for predictive solutions for simulating the spread of malaria. The structure incorporates sophisticated computing methods to improve the reliability of predicting malaria outbreaks. The study strives to provide a strong and effective tool for forecasting the propagation of malaria via the use of an AI-based recurrent neural network (RNN). The model is classified into two groups, consisting of humans and mosquitoes. To develop the model, the traditional Ross-Macdonald model is expanded upon, allowing for a more comprehensive analysis of the intricate dynamics at play. To gain a deeper understanding of the extended Ross model, we employ RNN, treating it as an initial value problem involving a system of first-order ordinary differential equations, each representing one of the seven profiles. This method enables us to obtain valuable insights and elucidate the complexities inherent in the propagation of malaria. Mosquitoes and humans constitute the two cohorts encompassed within the exposition of the mathematical dynamical model. Human dynamics are comprised of individuals who are susceptible, exposed, infectious, and in recovery. The mosquito population, on the other hand, is divided into three categories: susceptible, exposed, and infected. For RNN, we used the input of 0 to 300 days with an interval length of 3 days. The evaluation of the precision and accuracy of the methodology is conducted by superimposing the estimated solution onto the numerical solution. In addition, the outcomes obtained from the RNN are examined, including regression analysis, assessment of error autocorrelation, examination of time series response plots, mean square error, error histogram, and absolute error. A reduced mean square error signifies that the model’s estimates are more accurate. The result is consistent with acquiring an approximate absolute error close to zero, revealing the efficacy of the suggested strategy. This research presents a novel approach to solving the malaria propagation model using recurrent neural networks. Additionally, it examines the behavior of various profiles under varying initial conditions of the malaria propagation model, which consists of a system of ordinary differential equations.

## I. Introduction

Artificial intelligence (AI) has become increasingly prevalent for enhancing and optimizing mathematical modeling in various areas. Incorporating AI techniques into mathematical models results in a paradigm shift, allowing for more precise forecasting, faster computational and enhanced processes for making decisions. AI can aid in finding intricate connections between factors in mathematical simulations, which allows the creation of advanced and predictive algorithms. Artificial Intelligence’s capacity to deal with massive data sets and nonlinear relationships enables mathematical models to capture the nuances of actual-life events with better accuracy, whether or not in banking, engineering, or scientific research. The collaboration between AI and mathematical modeling not only speeds up research but also opens up fresh possibilities to resolve complicated problems that were previously thought to be computationally challenging.

Recurrent Neural Networks (RNNs) powered by AI have led to huge strides in medical classification, especially for forecasting diseases like malaria via mathematical modeling. RNNs in the use of healthcare data in sequence, such as medical records or time series data, to identify complex trends that may indicate the start of disease or advancement. Incorporating AI techniques improves RNNs’ prognostic abilities, resulting in more precise and timely diagnoses of health conditions. RNNs are especially well suited to the dynamic personality of malaria and the temporal nature of data about patients. Such models are capable of predicting the probability of malaria outbreaks or individual risks of infection by analyzing historical medical records, which include factors such as patient characteristics, clinical signs, and place of residence. The use of AI-based RNNs in medical prognosis not only aids in early identification of diseases, but also helps to customized treatment strategies.

The transmission of the pathogenic bacterium from one subject to another subject by various arthropods or parasites, such as mosquitoes, kissing bugs, tsetse flies, lice, sand flies, ticks, and so on, is what causes vector-borne diseases. Humans are one of the most common hosts of mosquito-borne diseases such as malaria, dengue, yellow fever, chikungunya, etc. [1]. One of the most complicated and deadly parasite illnesses is malaria. The parasite Plasmodium causes malaria, which spreads to people when infected female Anopheles mosquitoes bite them. In order to create eggs, female mosquitoes must consume blood. These eggs act as a bridge between the human and the bug, completing a cycle.

Over 228 million individuals have been infected with malaria, an endemic disease. According to the WHO [2], 405,000 individuals passed away in 106 countries and territories in 2018. Nigeria has the highest number of malaria infections and fatalities worldwide, making it a serious health issue. Because of its location, malaria posed a hazard to nearly 97% of Nigeria’s population. Only approximately 3% of Nigerians are thought to live in malaria-free areas, and this small number protects the other 3% [3]. In Nigeria alone, there are more than 100 million cases of malaria and more than 300,000 deaths annually; this number is more than the 215,000 HIV/AIDS deaths that occur there annually. KwaZulu Natal, Mpumalanga, and Limpopo are the three provinces in South Africa where malaria is still a problem; nevertheless, these provinces are recognized to be in various stages of malaria management and eradication [4]. One of the challenges to eliminate these areas has been found to be migration, as was most recently reported in the province of KwaZulu Natal, which records few local cases but a significant number of imported cases [5]. According to reports, the issue of border sharing with nations like Mozambique and Zimbabwe, where malaria rates are still prevalent, is a factor in the disease’s persistence in some areas of Limpopo [6].

Anopheles arabiensis, the primary malaria vector in rural areas, breeds in a variety of agricultural environments, including both permanent and temporary ground pools, especially in dry and semi-arid regions [7–11]. Anopheles stephensi is a potent carrier of P. vivax and P. falciparum. Up until 2011, the documented distribution of the disease was confined to a few South Asian nations and the majority of the Arabian Peninsula, except Saudi Arabia and Yemen in the southwest [12–14]. The first instance of invasive An. stephensi was identified in Djibouti in 2012 [15]. Since then, reports of its existence have come from Somalia (2019) [16, 17], Sri Lanka (2017) [18], Sudan (2016) [19], and Ethiopia (2016) [20]. The dynamic investigation of the varicella-zoster virus model in the framework of the Mittag-Leffler kernel has been examined by Qura Tul, Ain (2024) [21]. Qura Tul, Ain [22] (2022) examined the stochastic patterns of co-infections within a population with a finite carrying ability. The AR-Transform by Muhammad Akbar (2024) [23] is an innovative integral transformation prepared to reshape the field of computational mathematics and its applications.

**Fig 1:**
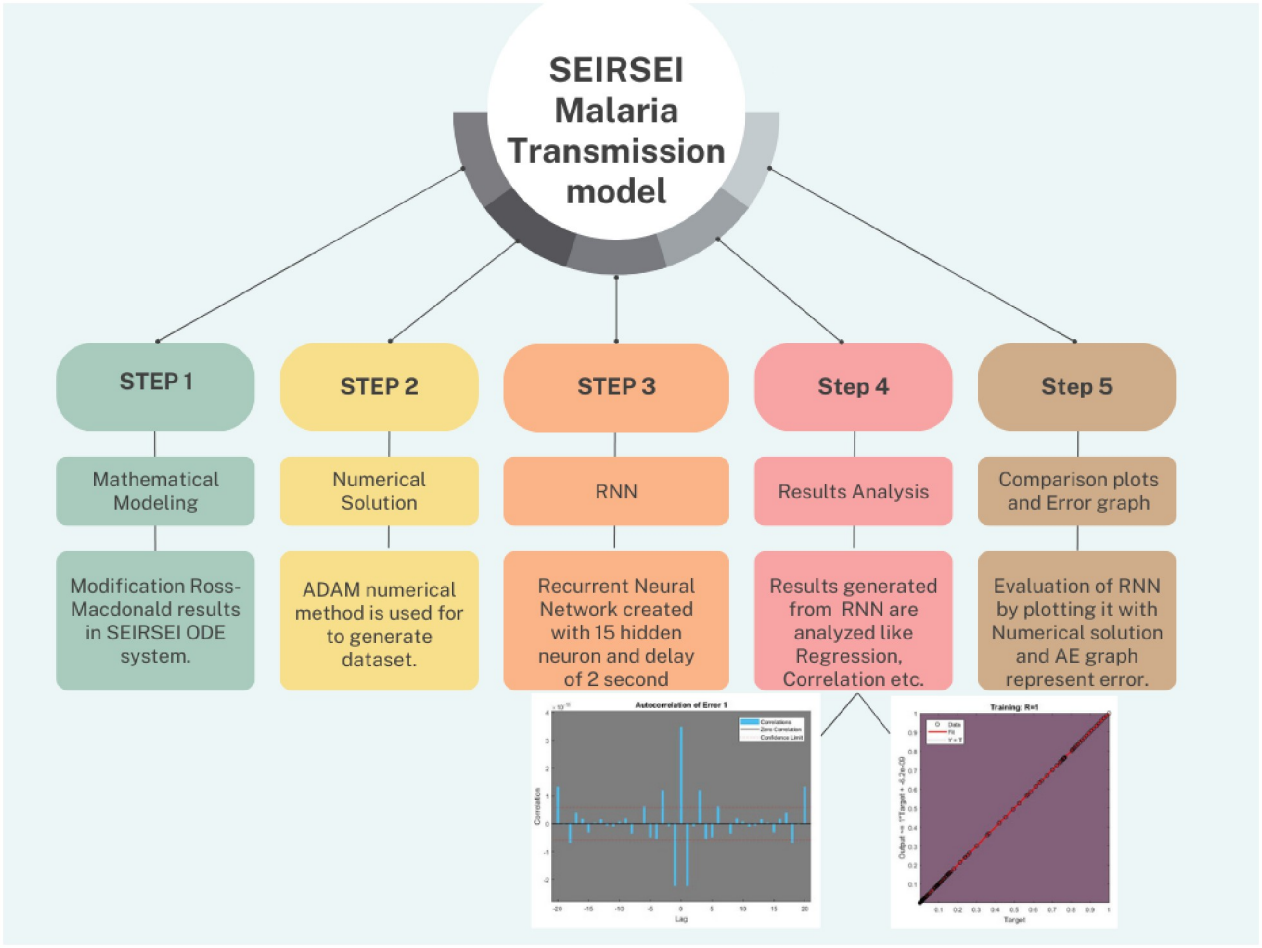
Workflow chart for SEIRSEI model.

Deep learning techniques have recently been shown to be the most successful method in medical diagnosis. They have been used to identify a wide range of disorders from biological images, such as Alzheimer’s disease [24] and breast cancer [25], COVID-19 [26], retinal hemorrhages [27], among others. Deep learning techniques can be used to separate leucocytes from blood smear images and categorize blood image samples into infected and uninfected data sets [28, 29]. Layers of connected units termed artificial neurons are used to create artificial neural networks (ANNs). An ANN is referred to as having a "shallow network" if it just has one input layer, one output layer, and possibly one hidden layer. The complexity of the network also grows as the number of levels does. Recurrent neural networks (RNNs), also known as ANNs with recurrent connections, are able to represent sequential data for sequence recognition and prediction [30]. High-dimensional, non-linear-dynamic hidden states make up RNNs [31]. The structure of hidden states serves as the network’s memory, and each hidden layer’s current state is dependent on its prior state [32]. RNN has many applications like Prediction problems [33], Speech Recognition [34], Text Summarization [35], Machine Translation [36] etc. For the current study the flow diagram is elaborated in Fig 1:

As the use of computers is getting common nowadays, different researchers combine its capability to solve complex problems with real life model. As artificial intelligence advances, the medical industry is changing as illnesses are now detected using RNN and the spread of an epidemic disease can be predicted using only data. Gorur [37] used shallow and RNN-based deep models, hospitalization status and gender recognition from arboviral medical records. Mamun [38] predicted Dengue Incidence in Bangladesh Using Machine Learning. Razzak [39] used RNN for malaria parasite classification. Kamble [40] studied detection of heart disease using an RNN deep learning network. Fujita [41] used RNN for prediction of Parkinson’s disease detection.

The current article is organized as follows:

- Section II represent the Mathematical modeling. A SEIRSEI ODE system is generated from Ross-Macdonald model.
- Section III presents the Solution methodology which includes techniques used in data preprocessing and algorithms used to solve the model.
- Results from RNN including visualization like Regression, histogram plots and table expressing statistical results are shown in Section IV.
- A minimal MSE signifies that the model’s estimates are more accurate.
- By getting a negligible or minimal Absolute error (AE) demonstrates the accuracy of the RNN computing technique.
- This research presents a novel approach to solving the malaria propagation model using recurrent neural networks.
- Section V concludes the study by presenting key insights of the study.

## II. Mathematical modeling

The Ross-Macdonald model is a dynamical system that is defined as the simplest basic model to explain malaria transmission [42].


$$\begin{array}{l} S_{H}\prime (y)+b\beta_{H}S_{H}(y)\frac{I_{M}(y)}{N_{H}}+dS_{H}(y)=dN_{H}+\gamma I_{H}(y),\\ I_{H}\prime (y)+(\gamma +d)I_{H}(y)=b\beta_{H}S_{H}(y)\frac{I_{M}(y)}{N_{H}},\\ S_{M}\prime (y)+\mu S_{M}(y)+b\beta_{M}S_{M}(y)\frac{I_{H}(y)}{N_{H}}=\mu N_{M},\\ I_{M}\prime (y)+\mu I_{M}(y)=b\beta_{M}S_{M}(y)\frac{I_{H}(y)}{N_{H}}. \end{array}$$ (1)

With initial conditions as:

$$S_{H}\left(0\right)=S_{0H},I_{M}\left(0\right)=I_{0M},I_{H}\left(0\right)=I_{0H},S_{M}\left(0\right)=S_{0M}.$$ (2)

Here, *S*_*H*_(*y*) and *S*_*M*_(*y*) stand for the number of susceptible people and mosquitoes at time respectively. The numbers of infected people and mosquitoes at time y are *I*_*H*_(*y*) and *S*_*M*_(*y*), respectively. The parameter b represents the frequency of mosquito bites, *β*_*H*_ represents the likelihood that an infected mosquito will infect a susceptible human, and *β*_*M*_ represents the likelihood that an infected human will infect a susceptible mosquito. Human recovery rate is parameter *γ*, whereas mosquito birth and mortality rates are parameters d and *μ*, respectively. The total populations of humans and mosquitoes, *N*_*H*_ and *N*_*M*_, are constant and are defined as follows:

$$S_{H}(y)+I_{H}(y)=N_{H},$$ (3)

And

$$S_{M}(y)+I_{M}(y)=N_{M}.$$ (4)

The exposed states for people and mosquitoes as well as the human recovery state are not taken into account in this model, which assumes that the number of populations is constant. Additionally, $b\beta_{H}\frac{I_{M}(y)}{N_{H}}$ and $b\beta_{M}\frac{I_{H}(y)}{N_{H}}$, which measure the force of infections in humans and mosquitoes, respectively, are linear regarding *I*_*M*_(*y*) and *I*_*H*_(*y*), and do not take the suffusing aspect of the infection into account. Due to these drawbacks Ross-Macdonald fails. The modified Ross-Macdonald consist of 4 classes for Humans and 3 classes for Mosquitoes describe as:

$$\begin{array}{c} S_{H}^{\prime }\left(x\right)+\frac{b\beta_{H}I_{M}\left(y\right)S_{H}\left(y\right)}{1+\nu_{H}I_{M}\left(y\right)}+\mu_{H}S_{H}\left(y\right)=\Lambda_{H}+\omega R_{H}\left(x\right),\\ E_{H}^{\prime }\left(y\right)+E_{H}\left(y\right)\left(\alpha_{H}+\mu_{H}\right)=\frac{b\beta_{H}I_{M}\left(y\right)S_{H}\left(y\right)}{1+\nu_{H}I_{M}\left(y\right)},\\ I_{H}^{\prime }\left(y\right)+\left(\mu_{H}+\gamma +\delta_{H}\right)I_{H}\left(y\right)=\alpha_{H}E_{H}\left(y\right),\\ R_{H}^{\prime }\left(y\right)+\left(\mu_{H}+\omega \right)R_{H}\left(y\right)=\gamma I_{H}\left(y\right),\\ S_{M}^{\prime }\left(x\right)+\frac{b\beta_{M}I_{H}\left(y\right)S_{M}\left(y\right)}{1+\nu_{M}I_{H}\left(y\right)}+\mu_{M}S_{M}\left(y\right)=\Lambda_{M},\\ E_{M}^{\prime }\left(y\right)+E_{M}\left(y\right)\left(\alpha_{M}+\mu_{M}\right)=\frac{b\beta_{M}I_{H}\left(y\right)S_{M}\left(y\right)}{1+\nu_{M}I_{H}\left(y\right)},\\ I_{M}^{\prime }\left(y\right)+\left(\mu_{M}+\delta_{M}\right)I_{M}\left(y\right)=\alpha_{M}E_{M}\left(y\right). \end{array}$$ (5)

With initial conditions as:

$$S_{H}(0)=k_{1},E_{H}(0)=k_{2},I_{H}(0)=k_{3},R_{H}(0)=k_{4},S_{M}(0)=k_{5},E_{M}(y)=k_{6},I_{M}(0)=k_{7}$$ (6)

In this case, the functions *E*_*H*_(*y*) represents the number of exposed humans, whereas *E*_*M*_(*y*) is depicts the mosquitoes at time y. The number of recovered people at time y is represented by the function *R*_*H*_(*y*). Let (Λ_*H*_, Λ_*M*_) show the birth rates which are included to the susceptible classes, and we assume that every newborn is healthy and susceptible. Later, mosquitoes and exposed people are shifted to the infected classes with the rates (*α*_*H*_, *α*_*M*_), when the latency periods for the vulnerable individuals who had previously transferred to the exposed states finish. For each category of people and mosquitoes, the natural mortality rates, or *μ*_*H*_, *μ*_*M*_, are specified. In addition to the natural mortality rates, the disease death rates (*δ*_*H*_, *δ*_*M*_) reduce the populations of diseased people and mosquitoes, respectively. The recovered class is infected by the sick individuals, who then lose their immunity and become vulnerable once more to the *ω* pace.

## III. Solution methodology

A holistic methodology has been proposed for solving the malaria problems with AI-based Recurrent Neural Networks (RNNs). The generated data set from ODEs is used for RNNs. The dataset is subsequently utilized for training the AI-based RNNs, enabling the model to acquire complicated periodic trends and connections within the data. The RNN design was picked for its capacity to detect consecutive relationships in time-series data, which is critical in comprehending the advancement of cases of malaria. Pay attention strategies within RNNs can also be incorporated to focus on pertinent characteristics and improve model comprehensibility Through the use of sophisticated AI innovations, the suggested approach strives at offering exact forecasts, aid in early finding, and make a contribution to the overall control and management of malaria outbreaks.

**Fig 2:**
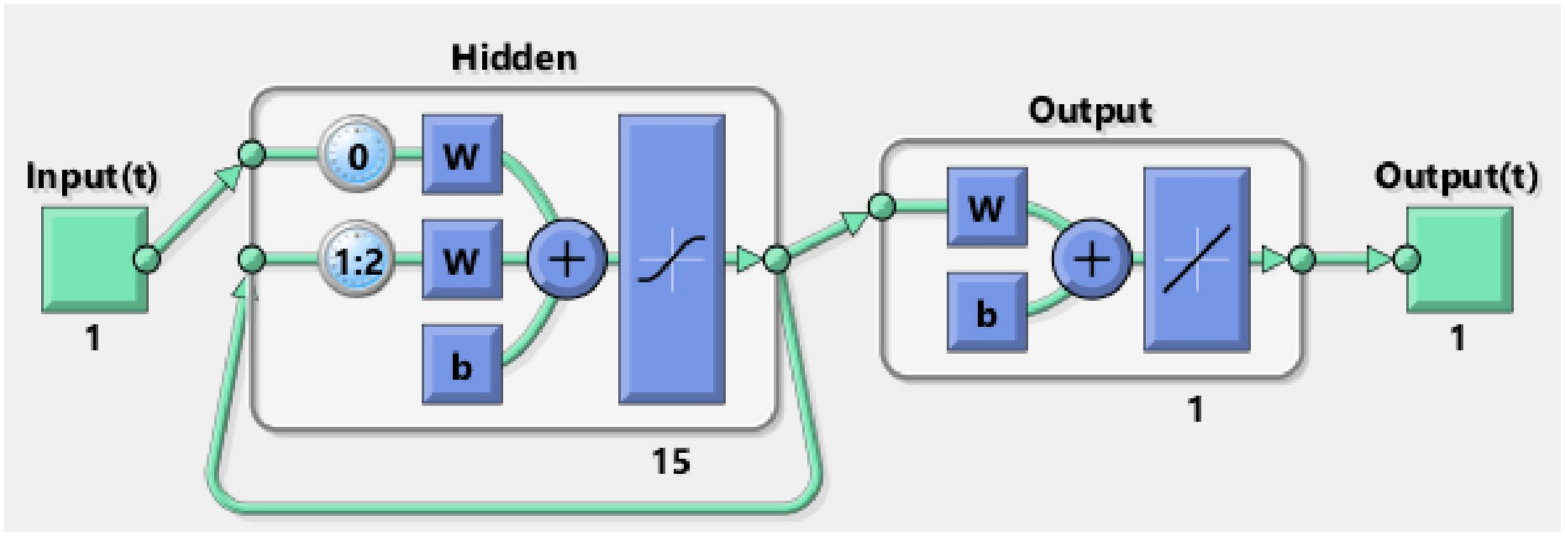
RNN workflow diagram.

The SEIRSEI model for malaria transmission consists of 7 nonlinear ordinary differential equations, 4 of them representing Susceptible, Exposed, Infected and Recovered human and 3 of the equations represents Susceptible, Exposed, Infected mosquito population. The model is analyzed using Recurrent Neural Network or RNN framework. The RNN requires a time series input and output. For input, a time series sequence of 0 to 300 days with step size of 3 days. For the current neural network, we used 15 hidden neurons and delay of 2 seconds. Fig 2 represents workflow RNN framework:

The numerical solution of ODEs is obtained by ADAM numerical method achieved using Mathematica’s ‘NDSolve’ command with input same as RNN i.e., 0 to 300 with step size of 3. The result is in form of a data points, last 5 rows of the dataset given in the Table 1.

**Table 1:** Dataset for 3rd variation of SEIRSEI model.

Feature 1	Feature 2	Feature 3	Feature 4	Feature 5	Feature 6	Feature 7
34.0486	0.0678264	1.71105	29.0097	1.45672	0.0249887	0.133351
34.1295	0.0669453	1.66326	28.966	1.47193	0.0245573	0.131102
34.2103	0.0660694	1.61699	28.9209	1.48706	0.0241321	0.128884
34.2912	0.0651991	1.57221	28.8742	1.50211	0.023713	0.126694
34.372	0.0643344	1.52886	28.8262	1.51708	0.0233001	0.124534

**Fig 3:**
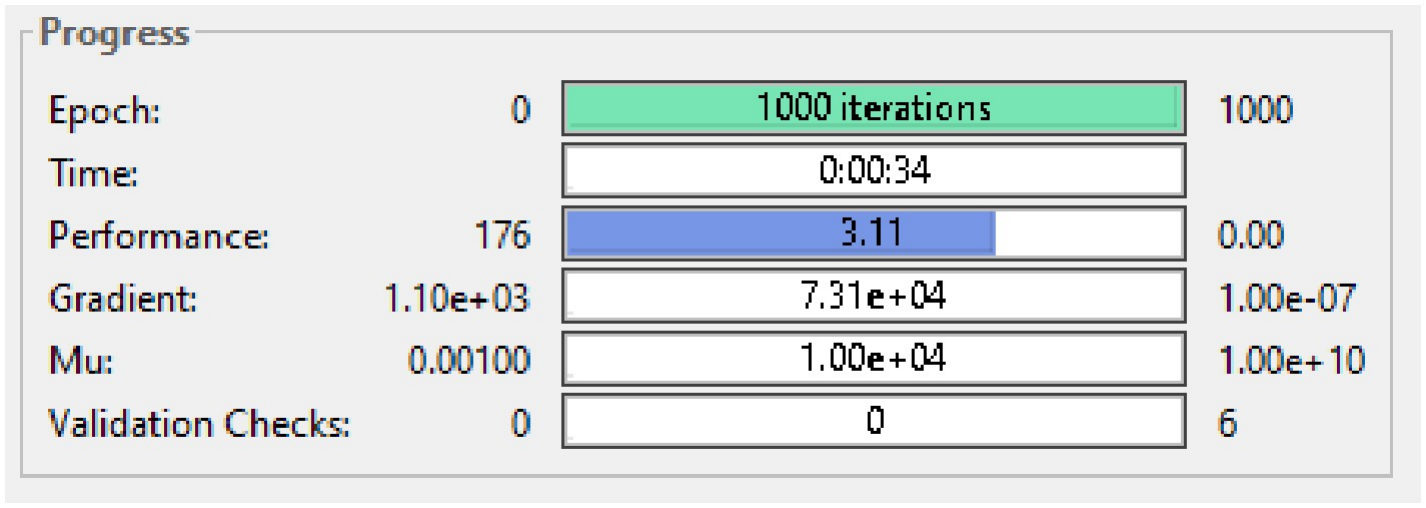
Statistical results for variation 3.

As we can see the dataset for one of the variations of SEIRSEI model Features 1 and 4 consist of overall large values than other features so change in any of feature (1 or 4) can cause big impact on the SEIRSEI model which ultimately results in bad RNN results. Fig 3 represents the training statistics for RNN on the aforementioned dataset.

As we can see in performance bar that for dataset RNN achieve performance of 3.11 which is basically Mean Square Error or MSE which is quite high. To fix the issue, a common data preprocessing method called Min-Max Normalization method is used. With this method, all scaled data between 0 and 1 is obtained. This can be done using the following formula:

$$x_{new}=\frac{x_{old}-x_{\text{min}}}{x_{\text{max}}-x_{\text{min}}}$$

**Fig 4:**
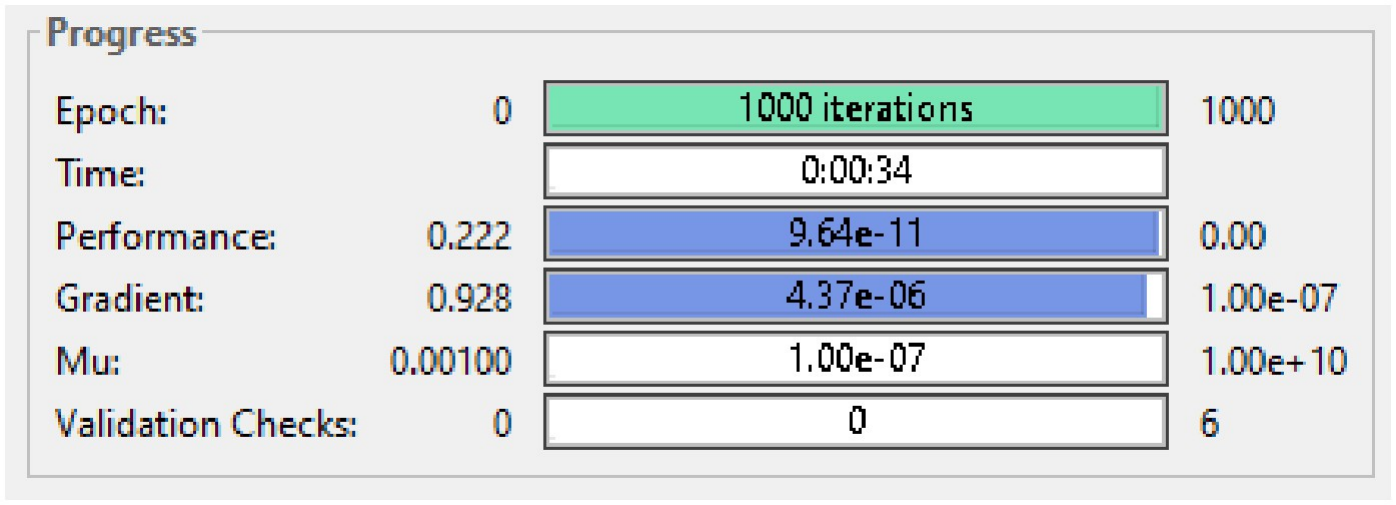
Results after preprocessing.

Where *x*_*old*_ is the value from dataset *x*_min_ is the minimum value present in the dataset and *x*_max_ is the maximum value present in the dataset. Using this technique RNN is able to achieve a MSE of 9.64E-11 (lower is better) as given in Fig 4.

For the given problem we taken in account 3 variations of initial conditions for ordinary differential equations to observe the impact on SEIRSEI profiles. Table 2 expresses value for each and Table 3 presents values of parameters which are constant throughout each variation.

**Table 2:** Variations for SEIRSEI model.

Variations	*k*_1_	*k*_2_	*k*_3_	*k*_4_	*k*_5_	*k*_6_	*k*_7_
**1**	10	8	3	1	1.3	0.6	0.4
**2**	20	16	6	2	2.6	1.2	0.8
**3**	30	24	12	3	3.9	1.8	1.2

Where *k*_*i*_, *i* = 1, 2, 3, …, 7 are initials conditions.

**Table 3:** Constant parameters.

Parameter	Value	Parameter	Value
Λ_*H*_	0.0027	*α*_*H*_	0.067
Λ_*M*_	0.027	*α*_*M*_	0.29
*β*_*H*_	0.1	*γ*	0.012
*β*_*M*_	0.3	*ω*	0.0011
*μ*_*H*_	0.0001	*ν*_*H*_	0.5
*μ*_*M*_	0.010	*ν*_*M*_	0.01
*δ*_*H*_	0.00002	*b*	0.01
*δ*_*M*_	0.05		

## IV. Results

This current research has successfully identified a total of 3 distinct variations in the initial conditions. A dataset of a time series sequence consists of values ranging from 0 to 300 and divided into one hundred intervals implies step size of 3, is inputted into the Recurrent Neural Network (RNN). Additionally, the RNN incorporates fifteen hidden layers and introduces a delay of two seconds after the numerical solution of Ordinary Differential Equations (ODEs) is established through the implementation of Adam’s numerical approach. The numerical solution is then used as output for RNN framework to train on. For aforementioned study the entire dataset is utilized to train the RNN framework. The results from RNN are evaluated and examined using multiple methods including correlation analysis, regression analysis, autoregression analysis etc. Table 4 below present different statistical results and Fig 1 represents the workflow of SEIRSEI malaria transmission model:

**Table 4:** RNN statistical results.

Variation	Performance	Gradient	MU	Time	Final iteration
**1**	3.48E-11	1.03E-05	1.00E-07	37s	1000
**2**	2.04E-11	0.000838	1.00E-07	38s	1000
**3**	9.64E-11	4.37E-06	1.00E-07	34s	1000

**Fig 5:**
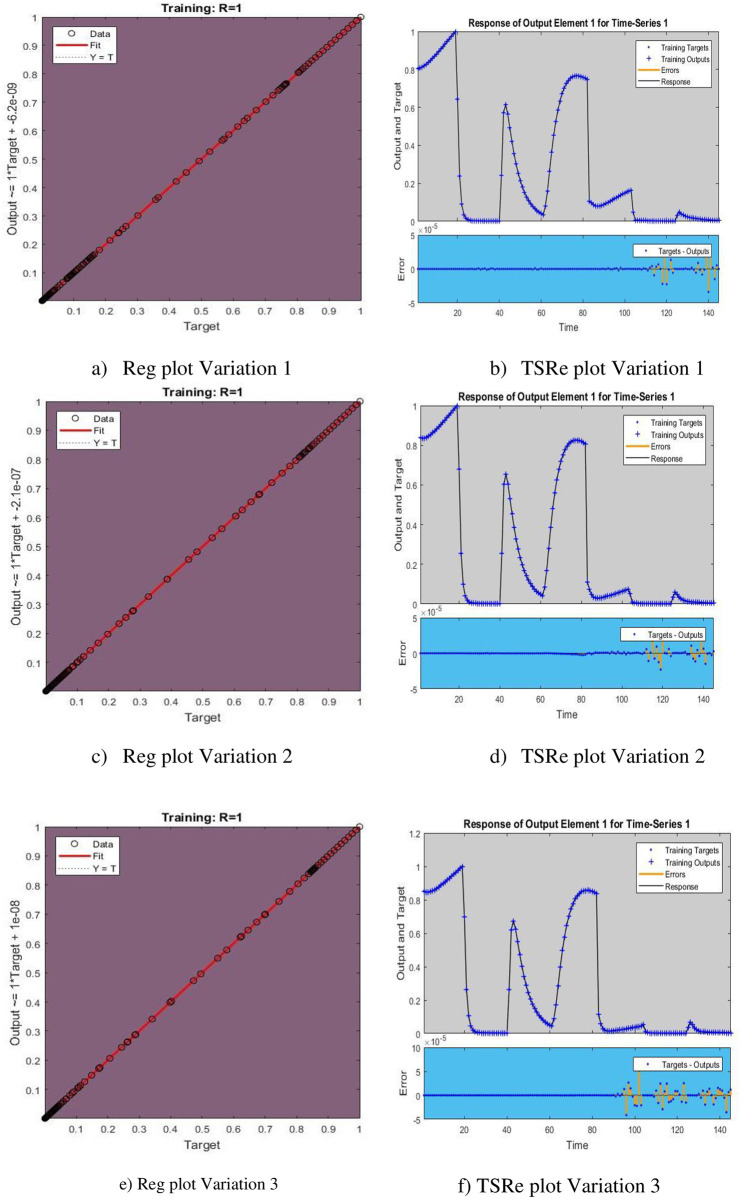
Regression (Reg) and Time Series Response (TSRe) plots. a) Reg plot Variation 1, b) TSRe plot Variation 1, c) Reg plot Variation 2, d) TSRe plot Variation 2, e) Reg plot Variation 3, and f) TSRe plot Variation 3.

**Fig 6:**
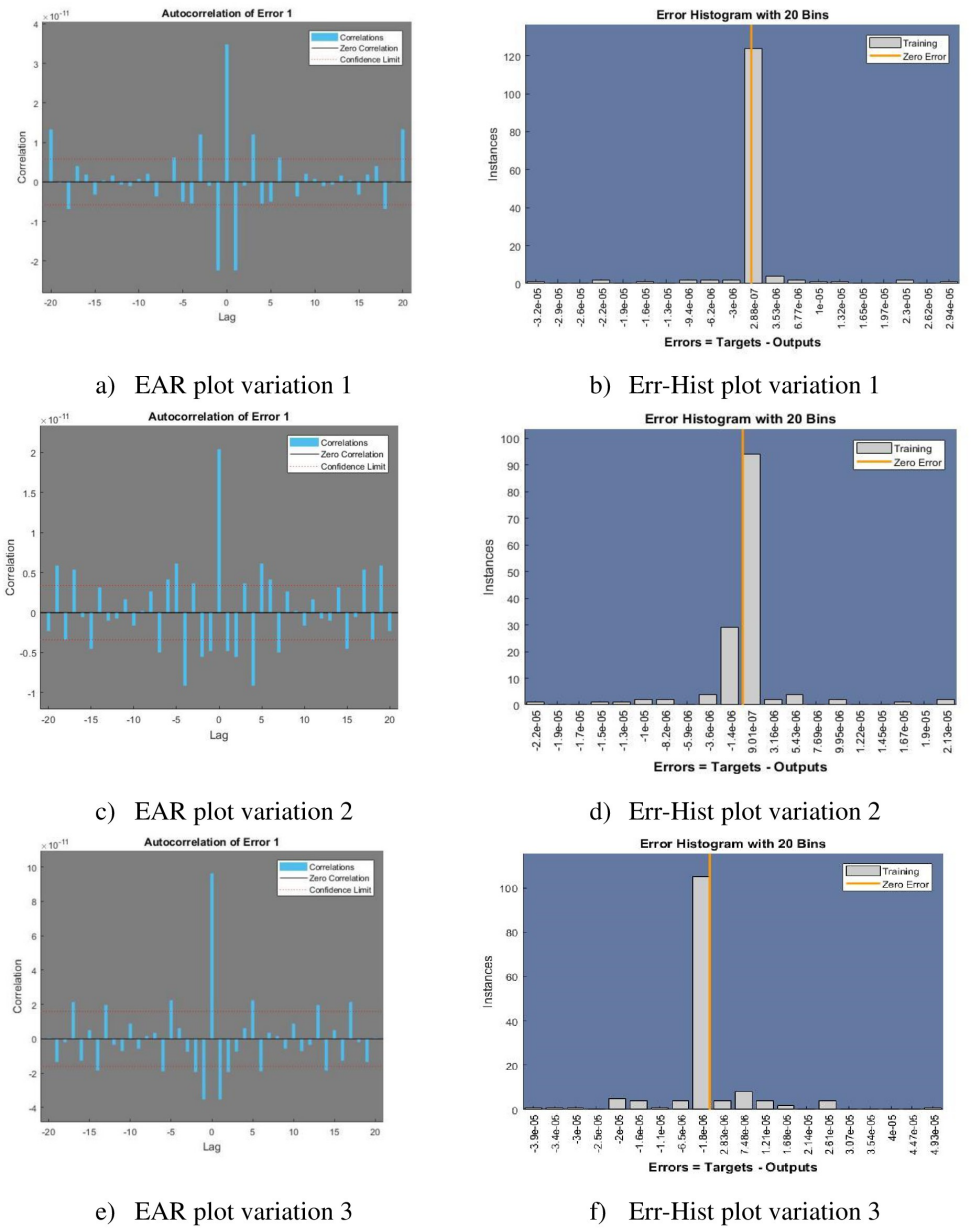
Error Autoregression (EAR) and Error Histogram (Err-Hist) plots. a) EAR plot variation 1, b) Err-Hist plot variation 1, c) EAR plot variation 2, d) Err-Hist plot variation 2, e) EAR plot variation 3, and f) Err-Hist plot variation 3.

**Fig 7:**
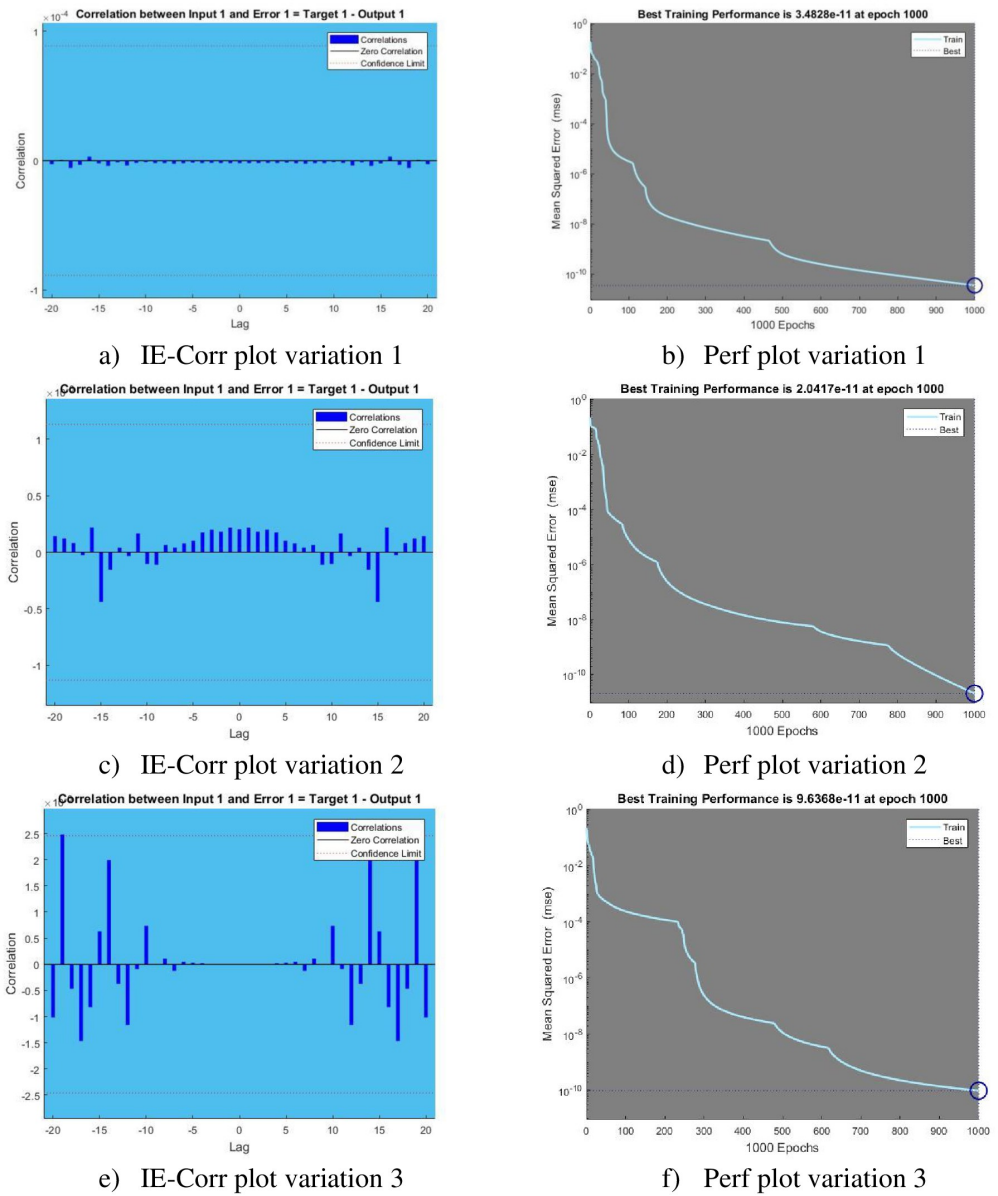
Input Error Correlation (IE-Corr) and Performance (Perf) plots. a) IE-Corr plot variation 1, b) Perf plot variation 1, c) IE-Corr plot variation 2, d) Perf plot variation 2, e) IE-Corr plot variation 3, and f) Perf plot variation 3.

Fig 5 represents the Regression and Time series response plots with Fig 6 representing error histogram and error autocorrelation. Performance and input error cross correlation are shown in Fig 7.

**Fig 8:**
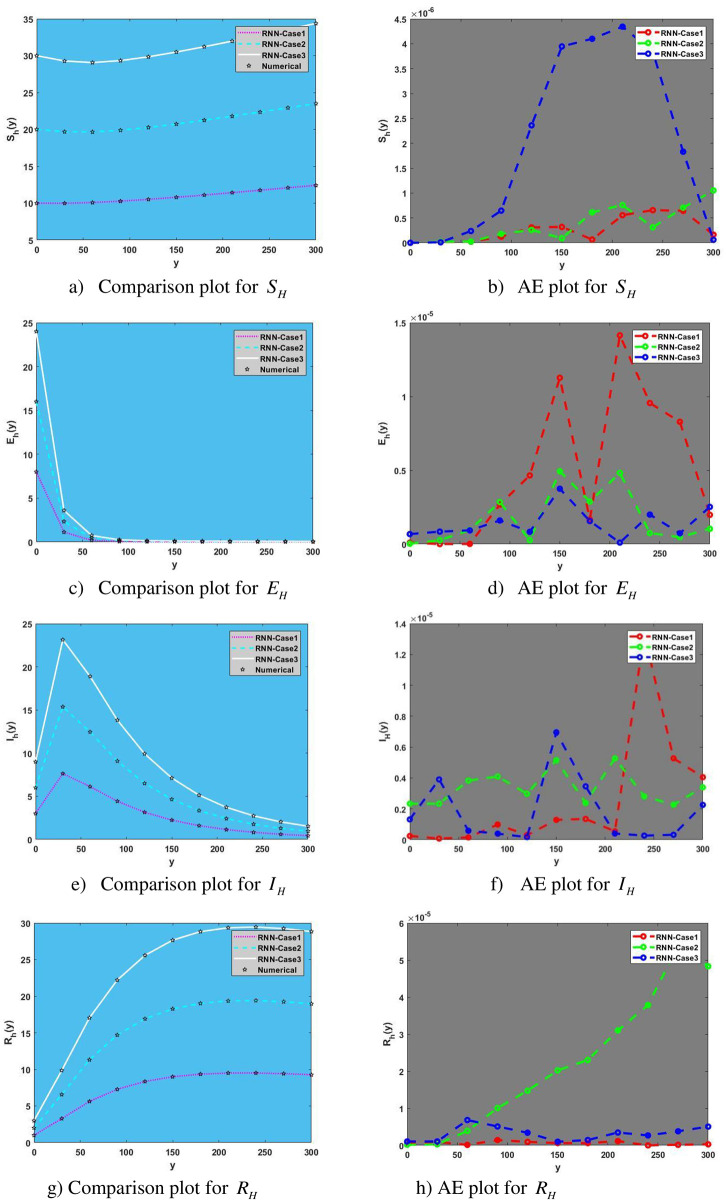
Comparison and Absolute Error (AE) plots for human population. a) Comparison plot for *S*_*H*_, b) AE plot for *S*_*H*_, c) Comparison plot for *E*_*H*_, d) AE plot for *E*_*H*_, e) Comparison plot for *I*_*H*_, f) AE plot for *I*_*H*_, g) Comparison plot for *R*_*H*_, h) AE plot for *R*_*H*_.

The comparison plot for human population is given in Fig 8 for Susceptible, Exposed, Infected and Recovered human population with AE plot representing absolute error between numerical solution and predicted solution using RNN at any value of time (y) from 0 to 300.

The time series response graph generated through Recurrent Neural Networks (RNNs) visually represents the model’s forecasts over an array of time steps. This graph shows the way the RNN conveys and determines temporal relationships in the data in real-time. An accurate RNN should show a precise match between forecast and observed values, demonstrating its ability to detect trends and patterns over time.

subfigures 5a, 5c and 5e represent regression plots for variation 1,2,3. The projected RNN solution displayed against the normalized numerical solution on the x and y axes, respectively, and the entire distribution is represented by a regression line. subfigures 5b, 5d and 5f show a time series response plot for variations 1, 2, and 3. The Time Series Response plot depicts the errors between a target time series (t) and an output time series (y) on the same axis.

Autocorrelation of error, which is frequently investigated in the field of time series analysis and regression analysis, refers to the study of connections between continuous errors or residuals in a dataset. When constructing prediction models, it is critical to determine if the errors show any systematic patterns over time. By tackling any identified trends or patterns present in the residuals, practitioners can refine their models by recognizing areas for enhancement while improving the model’s ability to predict.

The error autoregression plot for the variations 1, 2, and 3 is shown in subfigures 6a, 6c and 6e. When a signal or time series is compared to a delayed (lagged) version of itself, autocorrelation is used to determine the correlation between the two. subfigures 6b, 6d and 6f represent the error histogram. In which error feature of the dataset is plotted as histogram with 20 bins with instances representing the count for data points belonging to that class.

The Input Error Correlation measures the connection between the forecasting model’s errors or leftovers and the input variables used in the framework. comprehending if there’s a consistent trend or connection between the model’s findings inaccuracies and specific input features requires analyzing this correlation. A significant correlation may indicate that the model is not adequately capturing certain variables, resulting in systematic errors. Identifying and comprehending such correlations can help guide model refinement efforts, prompting the inclusion of new relevant features, adjusting model complexity, or addressing potential multicollinearity issues. The professionals may enhance the model’s precision as well as dependability by assessing the Input Error Correlation, ensuring that it adequately captures the complexity of the underlying data and improves the model’s predictive accuracy across multiple applications. The Mean Squared Error (MSE) is a critical metric for evaluating the efficacy of predictive models, especially in the framework of regression analysis. The main objective is to calculate the average squared difference between predicted and observed values. The MSE encapsulates the overall accuracy of the model across its predictions by taking the average of the squared residuals, where each residual is the difference between the model’s prediction and the true outcome. It is a useful tool for model evaluation and comparison because a lower MSE indicates a better fit between predicted and observed values. A reduced mean square error signifies that the model’s estimates are more accurate.

Input Error Correlation plots for variation 1,2,3 is shown in subfigures 7a, 7c and 7e. Using an input time series and an error time series, the cross-correlation of the inputs to the errors is plotted over a range of delays with Performance for each variation in given in subfigures 7b, 7d and 7f. Performance plot tells the behavior of Mean Square Error (MSE) as the number of iterations increases.

subfigures 8a, 8c, 8e and 8g expressing the comparison plot for human population with impact of different values of initial condition on population of Susceptible, Exposed, Infected, Recovered Human like from subfigure 8g we can see as we increase the value of initial conditions, population of recovered human also starting to increase. Absolute Error plots for different Initial condition are shown in Fig 8b, 8d, 8f and 8h with each figure representing AE plot for Susceptible, Exposed, Infected, Recovered Humans.

**Fig 9:**
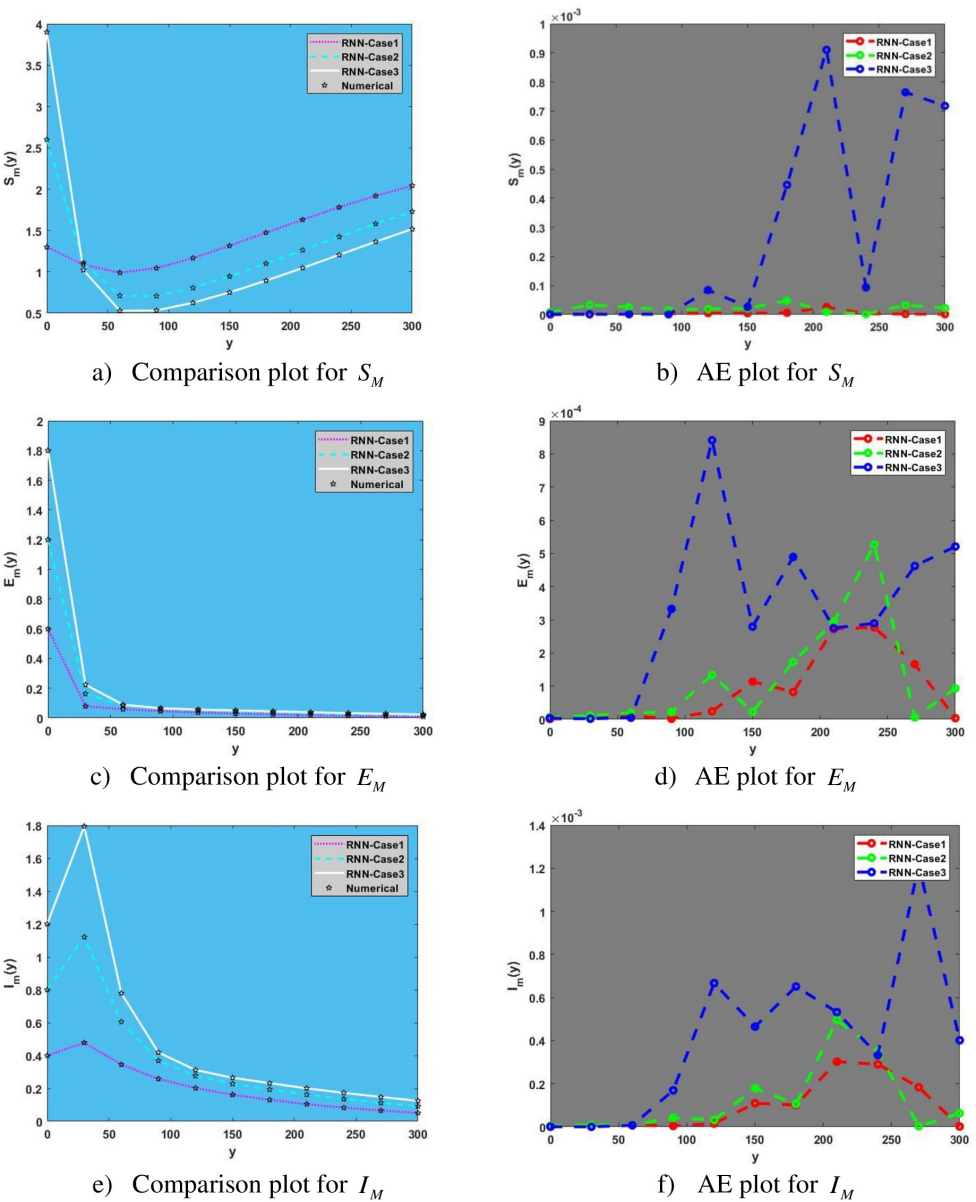
Comparison and Absolute Error (AE) plots for Mosquito population. a) Comparison plot for *S*_*M*_, b) AE plot for *S*_*M*_, c) Comparison plot for *E*_*M*_, d) AE plot for *E*_*M*_, e) Comparison plot for *I*_*M*_, f) AE plot for *I*_*M*_.

In Fig 9, subfigures 9a, 9c and 9e show a comparison plot for the human population with the effects of varying initial condition values on the population of susceptible, exposed, and infected mosquito.

The absolute error, an essential measure for evaluating the precision of forecasting techniques, is critical in reading the paper’s results. The absolute error provides a numerical indicator of the contrast between the model’s forecasts and actual observed values in the specifics of the study. The reported absolute error values deliver an obvious and apparent depiction of the model’s efficacy, as the paper strives to present novel perspectives or methodologies. By obtaining negligible absolute error, the model’s stability and occurance are demonstrated.

For example, Fig 8e shows that as initial condition values are increased, populations of infected mosquitoes also begin to rise. Subfigures 9b, 9d and 9f depict absolute error plots for various initial conditions and represent AE plots for susceptible, exposed, and infected mosquitoes, respectively.

## V. Conclusion

This paper presents the SEIRSEI malaria transmission model using the Recurrent Neural Network or RNN framework. In mathematical modeling the ODEs representing the model are classified in two distinct categories Human and Mosquito. The human category is further divided into 4 classes Susceptible, Exposed, Infected and Recovered with Mosquito category consisting only Susceptible, Exposed and Infected. Following are some conclusions from this research:

- For the SEIRSEI malaria transmission model, the recurrent neural network framework has been effectively developed.
- The RNN is trained using entire dataset with input in form of time series sequence from 0 to 300 days divided into 100 intervals.
- The RNN consists of 15 hidden neurons and delays of 2 seconds.
- The overlapping of the predicted solutions and Adams numerical solutions allows for the evaluation of the suggested RNN scheme’s correctness.
- This study presents a novel approach to solving the malaria propagation model using recurrent neural networks. Additionally, it examines the behavior of various profiles under varying initial conditions for the malaria propagation model consisting of system of ordinary differential equations.
- A reduced mean square error signifies that the model’s estimates are more accurate.
- The result is consistent with acquiring an approximate absolute error close to zero, revealing the efficacy of the suggested strategy. The minimal AE performances demonstrate the accuracy of the RNN computing technique.
- Through the Regression, Error Autoregression, Correlation, and time series response process, the suggested procedure’s consistency and dependability are evaluated in order to solve the SEIRSEI model.
